# The Olfactory Bulb in Newborn Piglet Is a Reservoir of Neural Stem and Progenitor Cells

**DOI:** 10.1371/journal.pone.0081105

**Published:** 2013-11-21

**Authors:** Lee J. Martin, Alyssa Katzenelson, Raymond C. Koehler, Qing Chang

**Affiliations:** 1 Division of Neuropathology, Department of Pathology, Johns Hopkins University School of Medicine, Baltimore, Maryland, United States of America; 2 Pathobiology Graduate Program, Johns Hopkins University School of Medicine, Baltimore, Maryland, United States of America; 3 The Solomon Snyder Department of Neuroscience Graduate Program, Johns Hopkins University School of Medicine, Baltimore, Maryland, United States of America; 4 Department of Anesthesiology, Critical Care Medicine, Johns Hopkins University School of Medicine, Baltimore, Maryland, United States of America; University of South Florida, United States of America

## Abstract

The olfactory bulb (OB) periventricular zone is an extension of the forebrain subventricular zone (SVZ) and thus is a source of neuroprogenitor cells and neural stem cells. While considerable information is available on the SVZ-OB neural stem cell (NSC)/neuroprogenitor cell (NPC) niche in rodents, less work has been done on this system in large animals. The newborn piglet is used as a preclinical translational model of neonatal hypoxic-ischemic brain damage, but information about the endogenous sources of NSCs/NPCs in piglet is needed to implement endogenous or autologous cell-based therapies in this model. We characterized NSC/NPC niches in piglet forebrain and OB-SVZ using western blotting, histological, and cell culture methods. Immunoblotting revealed nestin, a NSC/NPC marker, in forebrain-SVZ and OB-SVZ in newborn piglet. Several progenitor or newborn neuron markers, including Dlx2, musashi, doublecortin, and polysialated neural cell adhesion molecule were also detected in OB-SVZ by immunoblotting. Immunohistochemistry confirmed the presence of nestin, musashi, and doublecortin in forebrain-SVZ and OB-SVZ. Bromodeoxyuridine (BrdU) labeling showed that the forebrain-SVZ and OB-SVZ accumulate newly replicated cells. BrdU-positive cells were immunolabeled for astroglial, oligodendroglial, and neuronal markers. A lateral migratory pathway for newly born neuron migration to primary olfactory cortex was revealed by BrdU labeling and co-labeling for doublecortin and class III β tubulin. Isolated and cultured forebrain-SVZ and OB-SVZ cells from newborn piglet had the capacity to generate numerous neurospheres. Single cell clonal analysis of neurospheres revealed the capacity for self-renewal and multipotency. Neurosphere-derived cells differentiated into neurons, astrocytes, and oligodendrocytes and were amenable to permanent genetic tagging with lentivirus encoding green fluorescent protein. We conclude that the piglet OB-SVZ is a reservoir of NSCs and NPCs suitable to use in autologous cell therapy in preclinical models of neonatal/pediatric brain injury.

## Introduction

Regenerative medicine through novel cell-based therapies needs to be explored preclinically for treating perinatal brain damage [1,2]. The possibilities for cell-mediated neural repair after brain injury include recruitment of endogenous cells and transplantation of xenogenic, allogenic, or autologous cells. Recruitment of endogenous neural stem cells (NSCs) or neuroprogenitor cells (NPCs) for repair as well as endogenous neurogenesis after perinatal hypoxia-ischemia (HI) may have limited therapeutic benefit [3]; thus, cell transplantation is the alternative. To date, compared to adult models of central nervous system injury, relatively little work has been done on the transplantation of allogenic or xenogenic embryonic or postnatal stem cells, progenitor cells, or mesenchymal cells as a therapy in animal models of infant and childhood brain damage. In a neonatal mouse model of HI, retrovirally-transformed, immortalized, neonatal mouse cerebellum-derived stem-like cells (the C17.2 cell line) were transplanted into the cavitary lesion as a cell-polymer scaffold complex and were shown to engraft and differentiate into the three primary neural cell types and to integrate structurally [4]. In a neonatal rat model of HI, multipotent astrocytic NSCs from mouse forebrain subependymal zone differentiated into neurons at locations remote from the infarcted area [5]. Human cells have also been investigated [6-9]. A neonatal mouse model of excitotoxic brain damage has been used to evaluate the behavior of transplanted human embryonic germ (EG) cell-derived NSCs in the environment of an immature host forebrain that is injured [6]. Human EG cell derived-NSCs showed the ability to engraft, disseminate, differentiate, and replace neurons and oligodendrocytes in the damaged neonatal mouse brain. Human umbilical cord blood mononuclear cells protected against spastic paresis after intraperitoneal injection in the P7 rat model of HI [7]. Neonatal HI rats also showed attenuation of pathology and neurologic deficit with intracardial injection of human mesenchymal stem cells [8]. Human embryonic stem cell line H9-derived NSCs improved motor activity and enhanced cortical axon sprouting after transplantation into forebrain of neonatal HI rats [9]. The vast majority of preclinical work on cell therapy for neonatal brain injury has thus been done with rodents. A few studies have done transplantation of human EG cells [10] or sheep liver mesenchymal stem cells [11] in fetal sheep, but uninjured brain was the recipient. Importantly, more preclinical data needs to be collected using large animal models of neonatal brain damage to determine the translational relevance of cell therapy for neurologic restoration in neonatal and pediatric patients.

The concepts of neurogenesis and NSC niches in the postnatal mammalian brain have been building over the past century [12-16]. It is now believed that NSCs continually produce new neurons in the postnatal forebrain subventricular zone (SVZ). In rodents, the SVZ forms a cellular continuum with the core of the olfactory bulb (OB) through an extension called the rostral migratory stream (RMS) [12]. Cells that originate from the anterior SVZ migrate anteriorly within the RMS to reside within the OB. Thus, in rodents the OB core is considered to be the anterior-most part of the SVZ [14,17,18]. The complete SVZ-OB system is now generally considered a NSC niche [19,20], but the different components of the SVZ-RMS-OB system may have different properties and different potential values for cell therapy [21,22]. Scant work has been done on the characterization of the SVZ-OB NSC niche in large animals that are used in clinically relevant models of neonatal or pediatric brain injury. The objective of this study was to characterize using in vivo and cell culture methods the SVZ-OB NSC niche in the normal piglet as a prelude to autologous cell transplantation therapy for neonatal brain injury. We show that the newborn pig OB is a reservoir of NSCs and NPCs. 

## Materials and Methods

### Piglets

This study was carried out in strict accordance with the recommendation in the *Guide for the Care and Use of Laboratory Animals* of the National Institutes of Health. The protocol (SW09M37) was approved by the Johns Hopkins University Animal Care and Use Committee. All euthanasia was carried out with the animals under deep anesthesia with sodium pentobarbital, and all efforts were made to minimize animal stress and suffering. Male piglets were obtained from a local farm as done previously [23-27] at 1 day, 5-7 days and 30 days of age. The piglets were in the naïve state without brain injury. They were divided into 2 groups that were used for harvesting fresh tissue samples (n=6) or for histological studies (n=8) to characterize cell proliferation and NSC/NPC localizations. 

### In Vivo Cell Proliferation (DNA Synthesis) Assay

Cell proliferation in piglet brain was measured using the thymidine analog bromodeoxyuridine (BrdU) to track DNA synthesis during the S phase of the cell cycle [28] as previously done in rodents [20-22,29]. Piglets at 5 days of age (n=4) were injected with BrdU (Roche Molecular Biochemicals, Indianapolis, IN) (at 8 am, 50 mg/kg in normal saline, ip) and killed 24 h later and perfusion-fixed for brain retrieval. Age-matched piglets (n=4) without BrdU injections were controls.

### Biochemical studies of NSC/NPC markers in piglet brain

**Figure 1:**
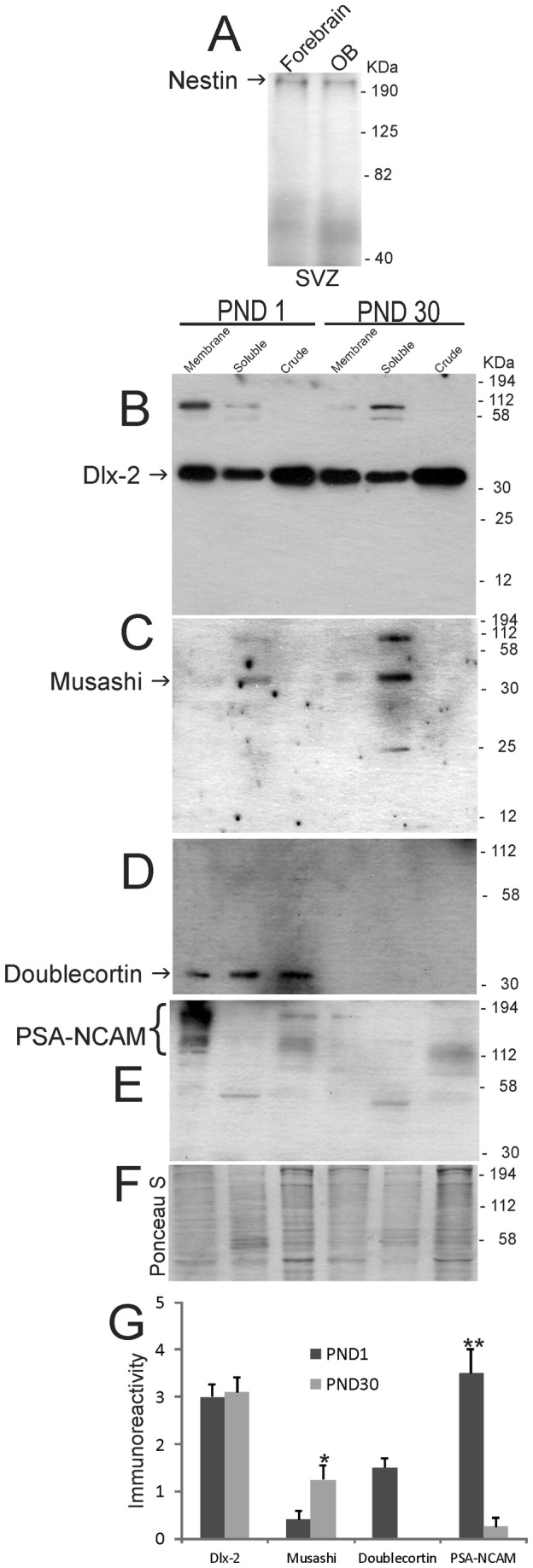
Western blots for neural stem cell and neuroprogenitor cell proteins in piglet forebrain. **A**. Nestin immunoreactivity in forebrain subventricular zone (SVZ) and olfactory bulb (OB) SVZ. Lower four blots show immunoreactivities for Dlx2 (**B**), musashi (**C**), doublecortin (**D**), and PSA-NCAM (**E**) in OB SVZ subcellular extracts (membrane, soluble and crude nuclear) at postnatal day (PND) 1 and PND 30. Molecular weight standards (in kDa) are shown at right side of the blots. **F**. Representative Ponceau S-stained nitrocellulose membrane is shown for protein loading. **G**. Graph showing the densitometry quantification of the levels of immunoreactivity for Dlx-2, musashi, doublecortin, and PSa-NCAM in membrane (Dlx-2 and PSA-NCAM) and soluble (musashi and doublecortin) fractions in the OB periventricular region at PND1 and PND30. Values are mean ± SD. Single asterisk denotes musashi PND30 vs PND1 p < 0.05. Double asterisk denotes PSA-NCAM PND1 vs PND30 p < 0.001.

Fresh brain samples from normal naïve piglets 1 day old (n=2) and 30 days old (n=2) were harvested for western blot experiments. Piglets received a lethal dose of sodium pentobarbital, were decapitated, and the brain with intact OBs was removed quickly and immersed in ice-cold phosphate-buffered saline (PBS). On ice, the brain was cut into 5 mm-thick coronal slabs from which the forebrain-SVZ and OB-SVZ were carefully microdissected under a surgical microscope. The dissections were done by the same individual (LJM) and were precise. A major advantage of a neonatal large animal model is that brain areas are larger and much more clearly divisible compared to neonatal mice. The brain samples were homogenized, fractionated, and subjected to SDS-PAGE as described [25,27]. Western blotting was done to detect in forebrain-SVZ and OB-SVZ (Figure 1) the expression of nestin, doublecortin (Dcx, a cytoplasmic microtubule-binding protein that directs migration of neuroblasts and newly born neurons), musashi (an RNA-binding protein that functions in posttranscriptional gene regulation), Dlx2 (a homeobox nuclear protein that functions in forebrain development), and polysialic acid neural cell adhesion molecule (PSA-NCAM, a cell membrane protein that functions in neuronal migration). The following primary antibodies were used: mouse monoclonal anti-nestin (Chemicon, Temecula, CA), rabbit polyclonal anti-Dlx2 (Chemicon), rabbit polyclonal anti-musashi (Chemicon), guinea pig polyclonal anti-Dcx (Chemicon), mouse monoclonal anti-PSA-NCAM (Chemicon). Immunoreactive proteins were visualized on nitrocellulose membranes with species-appropriate HRP-conjugated secondary antibodies and an enhanced chemiluminesence detection system (Pierce-Thermo Scientific, Rockford, IL). Western blots were analyzed quantitatively by densitometry using Bio-Rad Quantity One analysis software. Western blots were done in at least three independent experiments with different piglet brain samples and the fold change was calculated by dividing the optical density of each time-point by the loading control optical density. The protein loading controls were nitrocellulose membranes stained with Ponceau S. In developmental experiments, standard housekeeping proteins are unsuitable for protein loading because the levels of many of these proteins change during maturation and their enrichment differs in different subcellular fractions (Figure 1F).

### In vivo identification of BrdU, NSCs, NPCs, and newborn neurons in piglet forebrain

**Figure 2:**
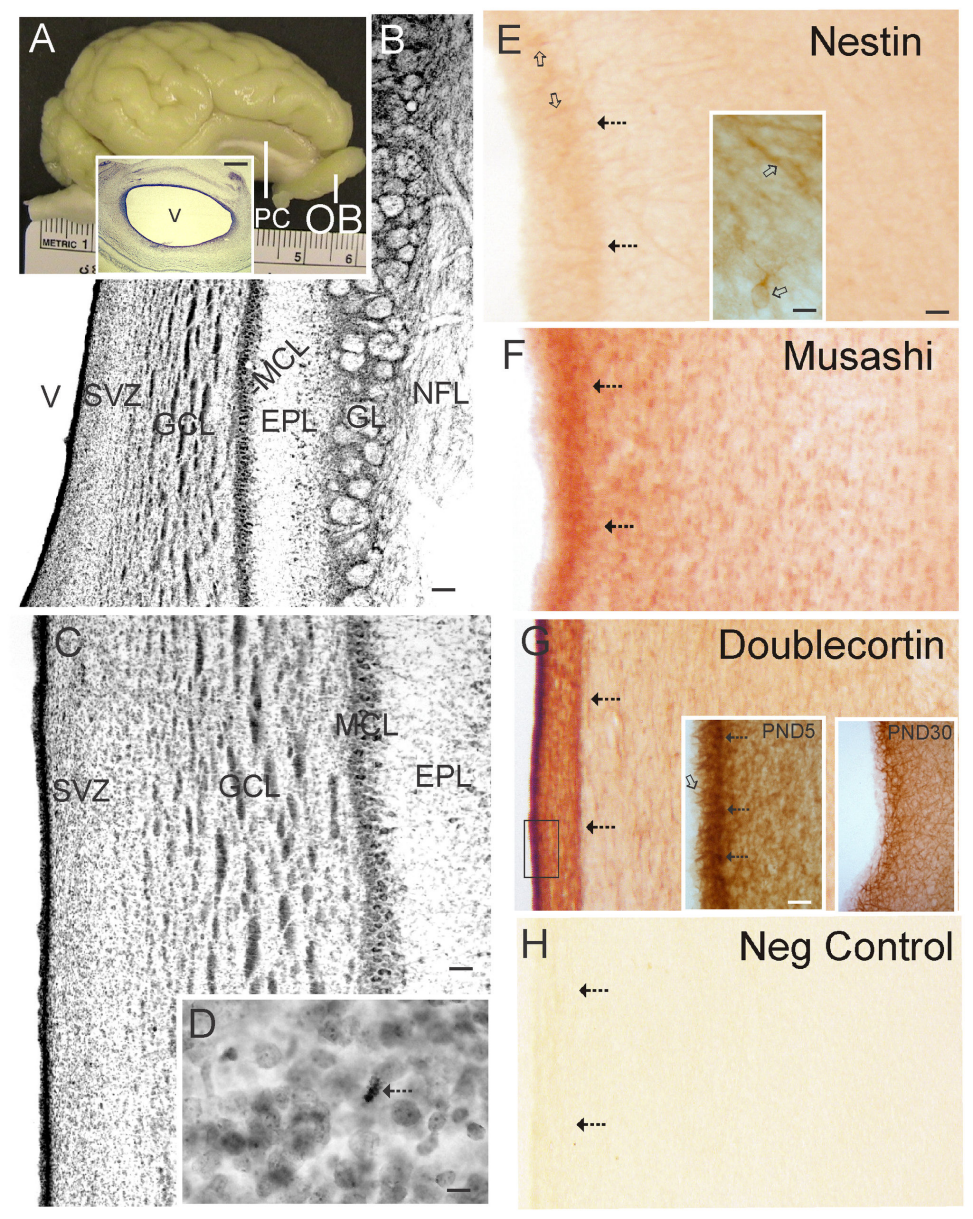
Piglet brain olfactory bulb (OB) gross anatomy, histology, and expression of neural stem cell and neuroprogenitor cell proteins. **A**. Gross neuroanatomy of the 5-day-old piglet brain showing the gyrencephalic cortex, primary olfactory cortex (PC, also known as piriform cortex) and OB. Inset shows a Nissl-stained transverse section through the OB revealing the patent cerebroventricular cavity (V) and prominent subventricular zone (dark blue lining of the ventricle) containing the ependymal and subependymal layers. Scale bar = 1.1 mm. **B**. Nissl-stained transverse section revealing the architectural lamination of the OB. From the ventricle (V) outward the layers are: the SVZ, the granule cell layer (GCL), the mitral cell layer (MCL, the mitral cells project to the primary olfactory cortex), the external plexiform layer (EPL), the glomerular layer (GL), and the nerve fiber layer (NFL) that originates from the neuroepithelium of the olfactory mucosa. Scale bar = 100 µm. **C**. Higher magnification image showing the cellular detail of the wall of the OB. Scale bar = 33 µm. **D**. Nissl staining reveals the presence of mitotic figures (arrow) in the SVZ. Scale bar = 8.3 µm. **E**-**G**. Immunohistochemical localization of nestin (E), musashi (F), and doublecortin (G) in the OB. Immunoreactivity is seen as orange-brown staining. Arrows identify the subependymal layer. Open arrows (in E) identify nestin^+^ cells. Inset in E shows nestin^+^ cells (open arrows) at higher magnification. The large nucleus (pale oval) and sparse cytoplasm with prominent neurites is typical of a NSC. Insets in G show doublecortin immunoreactivity at the ependymal/subependymal layers (see box in G) at higher magnification in piglet OB at postnatal day (PND) 5 and 30. In PND5 inset the open arrow identifies doublecortin^+^ processes extending into the ependymal layer and hatched arrows identify doublecortin^+^ cell bodies in the subependymal layer. Scale bar in E (same for F-H) = 33 µm. Scale bars for insets = 12 µm (E) and 20 µm (G) **H**. Nestin primary antibody was preadsorbed against nestin synthetic peptide for a negative control. No staining is seen.

**Figure 3:**
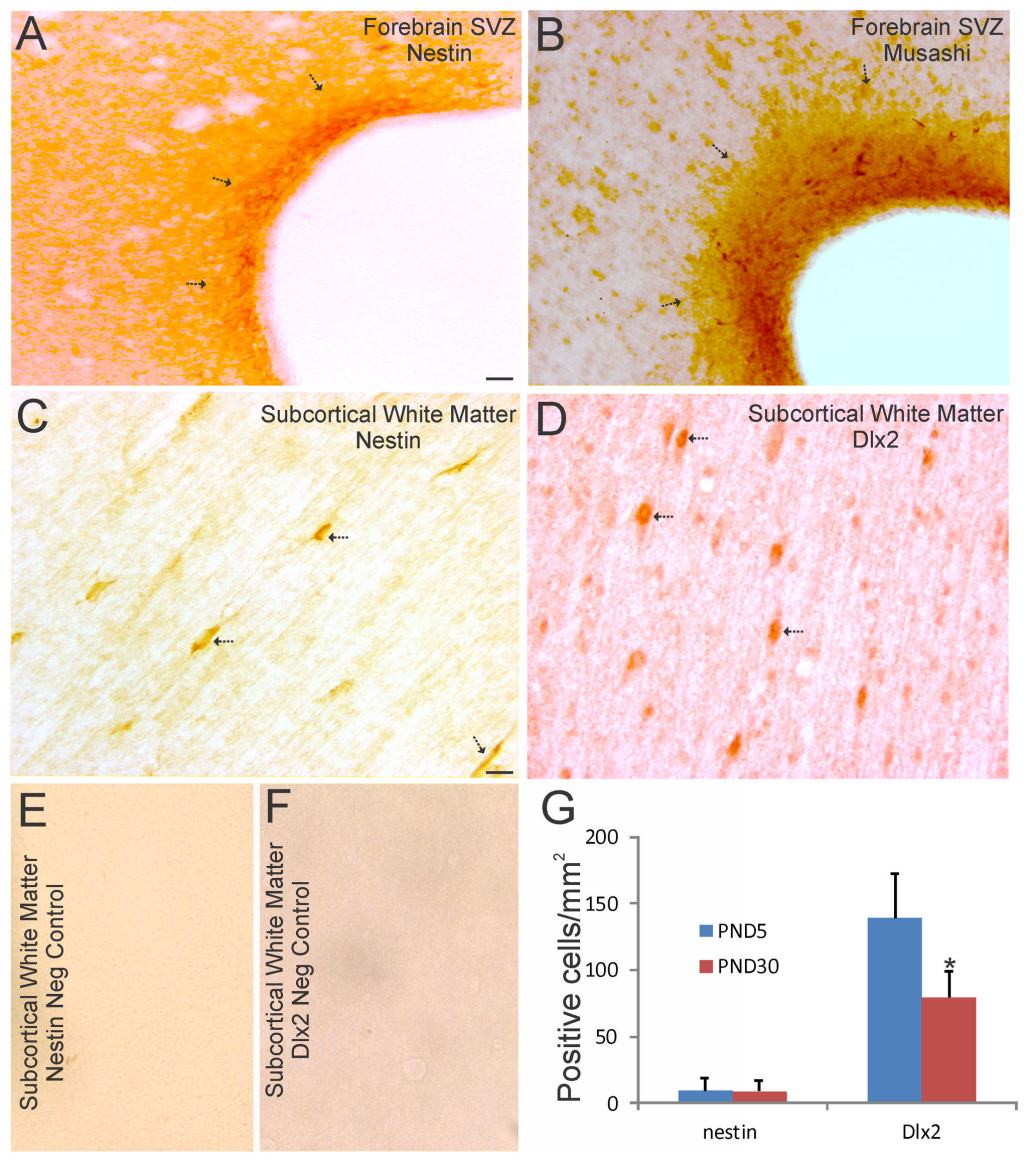
Localization of NSC and NPC markers in piglet forebrain SVZ and white matter. **A** and **B**. Immunohistochemical localization of nestin (A) and musashi (B) shows enrichment of these two NSC markers in piglet forebrain SVZ. Immunoreactivity is seen as orange-brown staining. Arrows identify the SVZ. The lateral ventricle is seen at right. The opaque layer at the interface of the SVZ and the ventricular cavity is the ciliated ependymal layer. Scale bar = 33 µm. **C** and **D**. Immunohistochemical localization of nestin (C) and Dlx2 (D), a neuroprogenitor marker, shows enrichment in individual cells (arrows) in piglet subcortical white matter. Scale bar in C (same for D-F) = 17 µm. **E** and **F**. Nestin primary antibody (E) was preadsorbed against nestin synthetic peptide, and Dlx2 primary antibody (F) was preadsorbed against recombinant Dlx2. No staining is seen in either preparation. **G**. Graph showing the number of nestin^+^ and Dlx2^+^ cell bodies in the subcortical white matter of the parasagittal gyrus in primary somatosensory cortex in piglets at postnatal day (PND) 5 and 30. Values are mean ± SD (n= 3-4 piglets/group). Asterisk denotes significant difference (p < 0.05) compared to PND5.

For histological analyses, piglets (6 piglets from BrdU experiments and 3 additional piglets at 5 and 30 days of age) received an overdose of sodium pentobarbital and were perfused through the heart with ice-cold PBS followed by 4% paraformaldehyde/PBS. The brains were cryoprotected in 20% glycerol/PBS and cut into sections on a sliding microtome at a thickness of 40 µm. Forebrain and OB sections from 5-7 day old piglets were used for cytological histology and immunolocalization of BrdU (cell proliferation assay) and cell-specific markers. Sections through the OB were stained with cresyl violet (Figure 2A-D). Sections through the forebrain and OB were stained immunohistochemically for nestin, musahi, Dcx, and β-tubulin III (Figures 2E-G, 3) using antibodies with specificities confirmed by immunoblotting with piglet brain extracts (Figure 1 and data not shown). Free-floating sections were permeabilized in 0.4% Triton-x 100, endogenous peroxidases were inactivated with H_2_O_2_, endogenous biotin was blocked using a commercial kit (Vector, Burlingame, CA), then sections were further blocked in 10% normal goat serum or normal donkey serum followed by incubation in primary antibody for 48 hours. For single-marker labeling, immunoreactive proteins were visualized with species-appropriate secondary antibodies and avid-biotin detection using diaminobenzidine (DAB) as chromogen. Negative control experiments on piglet brain sections were done with primary antibodies preadsorbed with recombinant proteins (Figures 2H,3E,F). 

Immunofluorescence was used for double-marker labeling within the same section. BrdU was detected with a rat monoclonal antibody (BD Bioscience, San Jose, CA) in combination with rabbit polyclonal antibodies (Dako, Carpinteria, CA) to glial fibrillary acid protein (GFAP) for astrocytes, rabbit polyclonal antibodies (Chemicon) to oligodendrocyte-2 transcription factor (Olig2) for oligodendrocytes, guinea pig polyclonal antibody to Dcx, rabbit polyclonal antibodies to β-tubulin III (Covance), or mouse monoclonal antibody (Chemicon) to the neuronal nuclear matrix protein (NeuN) for neurons. Species-specific secondary antibodies conjugated to Alexa Fluor 488 or Alexa Fluor 594 (Invitrogen Corporation, Carlsbad, CA) were used for visualization. Sections were imaged using a Zeiss LSM 510 confocal microscope. 

Counting of immunolabeled cells was done by profile counting. DAB-positive (+) cells for nestin and Dlx2 were clearly divisible at 400x magnification and were counted in the subcortical white matter of primary somatosensory cortex (AP +20 mm, L 12.5 mm) [23] in six sections from each piglet. Cells were counted in at least 6 different non-overlapping microscopic fields in each section. Immunofluorescent cells that were BrdU^+^ or BrdU^+^/GFAP^+^, BrdU^+^/NeuN^+^, BrdU^+^/Olig2^+^, and BrdU^+^/Dcx^+^ were counted at 1000x magnification in the OB-SVZ and forebrain-SVZ (AP +20 mm, L 5-7.5 mm) in four sections from each piglet. Cells were counted in at least 10 different non-overlapping microscopic fields in each section. 

### Isolation and culture of NSCs from piglet forebrain-SVZ and OB-SVZ

Fresh primary tissue samples from normal naïve postnatal day (PND) 5 piglets (n=2) were harvested for cell culture experiments. Piglets received a lethal dose of sodium pentobarbital and were decapitated. The entire brain was removed quickly and immersed in ice-cold Hanks balance salt solution (HBSS). The cerebrum was cut into 5 mm-thick coronal slabs from which the forebrain-SVZ and OB-SVZ were carefully microdissected under a Zeiss surgical microscope. Forebrain-SVZ samples were collected from the residual ganglionic eminence at the dorsolateral notch of the lateral ventricle adjacent to the striatum and corpus callosum. OB-SVZ samples were collected by circumferentially cutting ~0.25 mm of periventricular tissue within the OB (Figure 2A inset). These tissue samples contain primary cells and cells that potentially possess features of NSCs and NPCs, including multipotency, self-renewal, self-maintenance, expression of NSC and newborn neuron proteins, and responsiveness to NSC-trophins such as FGF2 [20,29]. 

The SVZ tissues were digested in 0.25% trypsin-EDTA and incubated (37°C, 5% CO_2_ and 95% air) for 20 min followed by trituration to dissociate cells. Cells were seeded on poly-D-lysine coated 35-mm well tissue culture plates at a density of 6x10^5^/ml with DMEM (Gibco/Life Technologies, Rockville, MD) containing high glucose, glutamine, FGF2, B27 (Gibco), DNase I and antibiotics (100 U/ml penicillin and 100 μg/ml streptomycin). After 24 h, the medium contained non-adherent, floating clonal colonies of cells that are called neurospheres. 

Primary neurospheres were pelleted, resuspended, dissociated by trypsin digestion and trituration. Cell suspensions were transferred to non-coated wells for further expansion of highly proliferating sphere-forming cells. After 9 passages of the original primary spheres, floating spheres were allowed to grow in poly-D-lysine coated 35-mm well plates in DMEM containing high glucose, glutamine, and supplemented with B27 and antibiotics. These spheres were diluted, by a limited dilution method [20,21], to isolate individual spheres and were transferred into individual wells with neurosphere growth medium for an additional passage. Neurospheres were then collected and cryopreserved in DMEM containing DMSO and fetal bovine serum. 

Piglet forebrain-SVZ and OB-SVZ neurosphere multipotency was confirmed using single cell-clonal analysis of neurospheres derived originally by limited dilution (1 cell/100 μl in wells of a 96-well plate) of dissociated primary neurosphere cells as described for rodents [20,21]. The neurospheres were allowed to attach to the culture plates and then were cultured for an additional 3 days. The attached neurosphere cultures were fixed with 4% paraformaldehyde and stained with antibodies to localize markers for astrocytes (GFAP), neurons (β-tubulin III), and oligodendrocytes (the sulfatide O-antigen O4) as described [20,21,27,29]. Triple labeling was done using primary antibodies from different species of animals or different immunoglobulin subtypes. The primary antibodies used were from commercial sources: mouse monoclonal anti-GFAP IgG (Roche, Indianapolis, IN), rabbit polyclonal anti-GFAP IgG (Dako), rabbit polyclonal anti-β-tubulin class III IgG (TuJ1 antibody, Covance, Richmond, CA) or MAP2 IgG (Sigma, St. Louis, MO), and mouse monoclonal anti-O4 IgM (Chemicon). Secondary antibodies or avidin (Invitrogen) were conjugated to Alexa-488 (green), Texas Red (red) or Cascade Blue (blue). Controls for antibody specificity were done for each antibody by incubating cultures in the primary antibody IgG isotype instead of primary antibody.

Isolation and purification of piglet forebrain-SVZ and OB-SVZ NSCs was done by immunopanning for notch-1 receptor known to be a mammalian NSC marker [30]. An antibody that detects an extracellular epitope on notch-1 was used (Proteintech, Chicago, IL). For direct immunopanning, notch-1 antibody was directly coated on the culture wells and dissociated neurosphere cell suspensions were applied to the wells after blocking in suspension with 0.5% BSA, followed by washing and retrieval of immunoisolated cells by trypsinization and centrifugation. 

### OB neurosphere growth responses

For neurosphere growth experiments as done before [29], cryopreserved neurospheres were thawed and expanded in vitro again. After one passage, they were collected by spinning, washed with HBSS once and dissociated with TryLE Express (Gibco) for 10 min at 37°C and triturated into single cell suspensions. Neurosphere-derived cells were seeded in serum-free medium (F12/DMEM) containing 20 ng/ml FGF2 (R&D Systems, Minneapolis, MN) and epidermal growth factor (EGF, R&D Systems) at a cell density of 10^5^/ml. Quantification was done by counting neurospheres in at least 9 randomly selected fields using a grid. Total numbers of neurospheres at 24 h of stimulation from 4-6 wells (33 mm) were averaged and compared. Neurospheres were allowed to attach and the number of cells per neurosphere was estimated. Phase contrast photographs were taken with a Nikon inverted microscope. 

### Lentiviral transfection of OB-SVZ NSCs

Neurosphere-forming OB-NSCs isolated from piglet were stably transfected with a GFP reporter gene using lentivirus so that these cells could be identified in future experiments. A lentiviral expression system for GFP was engineered [22] using a ViraPower kit (Invitrogen, Carlsbad, CA). A lentiviral-based expression vector containing the GFP coding sequence, PLL3.7, and three other plasmids, pLP1, pLP2 and pLP/VSVG (Invitrogen) were co-transfected using Lipofectamine 2000 into 293FT producer cells (Invitrogen), after 3 passages, at a density of 1.2 x 10^6^/ml in growth medium (without serum and antibiotics) in cell culture flasks. After 14 h of incubation at 37°C, the medium containing lipofectamine was removed and replaced with DMEM containing 10% FBS, 2 mM L-glutamine, 0.1 mM MEM non-essential amino acids, 1% penicillin/streptomycin, and 1 mM MEM sodium pyruvate. Supernatants were harvested at 48 hs post-transfection by removing medium into 15 ml sterile tubes, which were then centrifuged at 3000 rpm for 15 min at 4°C to pellet and remove cell debris. Viral particles were further purified and concentrated by centrifugation at 50,000 rpm. The pellets were resuspended in sterile PBS. Titer assays were done by transfecting 293FT cells with a series of concentrations of lentivirus preparations (mock and 10^-2^-10^-6^ dilutions in six-well plates) and then counting cell colonies that were GFP^+^ by fluorescence microscopy. Piglet OB-SVZ neurospheres were used for transfection after at least 9 passages. For OB-NSC transduction, a 5 µl aliquot of 10^9^ viral particles was added into each neural sphere culture well (in 35 mm wells with the same medium used for 293FT cell post-transfection culture). They were screened for GFP fluorescence after 48 hours (~68% spheres were green at 48 hours after exposure to lentivirus). Mock transfected neurospheres did not have green fluorescence. The spheres were harvested by centrifugation at 900 rpm for 5 min and then were resuspended in PBS and were cryopreserved.

### Statistical analysis

Western blot densitometry measurements, cell counts, and neurosphere measurements were used to determine group means and variances. Comparisons among groups were analyzed using a one-way analysis of variance and a Newman-Keuls post-hoc test. 

## Results

### Expression and localization of NSC/NPC proteins in piglet brain

Western blotting was used to detect NSC and NPC proteins in newborn piglet brain fractions and potential changes in these proteins in early postnatal development (Figure 1). We used antibodies to nestin, musashi, PSA-NCAM, Dlx2, and Dcx. Nestin is a marker for NSC and NPCs [31-34]. Nestin was detected as a single band at the predicted size of ~220 kDa in crude extracts of microdissected forebrain-SVZ and OB-SVZ (Figure 1A). Dlx2 is a distal-less homeobox transcription factor gene product essential for forebrain development [35] as well as specification and tangential migration of interneurons to the OB [36]. Dlx2 was detected as a prominent band at ~34 kDa in fractions of OB-SVZ (Figure 1B) consistent with other work [37]. Dlx2 was found at greatest levels in crude nuclear fractions (Figure 1B) as expected for a transcription factor [35-37]. The levels of 34 kDa Dlx2 did not change substantially in piglet OB-SVZ extracts at PND1 compared to PND30 (Figure 1A,G). Musashi is a 35 kDa RNA-binding protein that plays a role in asymmetric cell division and regulates NSC self-renewal [38]. Musashi was detected specifically in the soluble fractions of piglet OB-SVZ at ~35 kDa (Figure 1C), and the levels increased as the brain matured (Figure 1C,G). Dcx is a 45 kDa microtubule-associated protein that is expressed specifically in most migrating newly born neuroblasts [39]. Dcx was detected as a highly specific band at ~45 kDa in all subcellular fractions in PND1 piglet OB-SVZ region (Figure 1D); the levels were down-regulated prominently by PND30 (Figure 1D,G). With long exposures during development of western blots, Dcx was detected faintly in the OB-SVZ soluble fraction at PND30 (Figure 1D). The polysialated form of NCAM is a highly post-translationally modified cell surface glycoprotein that functions to abrogate intercellular adhesion and is a NPC marker [40]. In piglet brain, PSA-NCAM was found as a broad immunoreactive band (~130-200 kDa) at very high levels in enriched plasma membrane fractions of OB periventricular region at PND1 (Figure 1E). The size and broadness of this immunoreactive band of proteins is consistent with PSA-NCAM and indicative of a high level of post-translational modification [41]. By PND30, the PSA-NCAM levels were down-regulated and appeared less post-translationally modified because of the diminished broadness of the band (Figure 1E,G). 

The OB in the 5-day-old piglet is a prominent ~1.5 cm-long structure that protrudes from the ventral frontal lobe (Figure 2A). The piglet OB has a patent cerebroventricular cavity, unlike the OB in mouse and rat [20]. Histologically (Figure 2B,C), the lamination of the piglet OB is divisible into 6 layers, including the SVZ, granule cell layer (GCL), mitral cell layer (MCL), external plexiform layer (EPL), glomerular layer (GL), and olfactory nerve fiber layer (NFL). The OB SVZ layer is ~0.5 mm in thickness (Figure 2B,C) and contains mitotic figures (Figure 2D). The GCL is seen with large islands of granule neurons (Figure 2B,C). The MCL is the narrowest layer and contains large neurons (Figure 2C) that receive axonal innervation from olfactory mucosa receptor cells onto their dendrites located in the GL (Figure 2B) and dendro-dendritic synapses from granule cells [42].

Immunohistochemistry was used to localized NSC and NPC markers in piglet OB and forebrain sections (Figures 2,3). The piglet OB-SVZ was modestly positive for the NSC and NPC marker nestin [31-34] (Figure 2E). While nestin immunostaining was mostly uniform throughout the ependymal and subependymal layers, subsets of individual nestin^+^ cells were observed in the subependymal layer (Figure 2E, inset) but not in the ependymal layer. Although much lightly-staining immunoreactivity for nestin was localized diffusely in the neuropil (Figure 2E), this staining was specific as determined by nestin antibody preadsorption controls (Figure 2H). In contrast, the OB-SVZ was strongly positive for musashi (a NSC marker) [38] and Dcx (a neuroblast marker) [39] (Figure 2F,G). Musashi^+^ cells were concentrated in the subependymal layer but not in the ependymal layer (Figure 2F). Dcx immunoreactivity was also concentrated in the subependymal layer (Figure 2G). Individually divisible Dcx immunoreactive cell bodies and processes were observed in the SVZ (Figure 2G, left inset). These Dcx^+^ processes were contiguous with or penetrated the overlying ciliated ependymal cell layer (Figure 2G, left inset). In the OB-SVZ at PND30 (Figure 2G, right inset), Dcx immunoreactivity was attenuated compared to PND5 piglets (Figure 2G, left inset). This immunohistochemical result is consistent with the western blot results demonstrating an attenuation of Dcx immunoreactivity with maturation (Figure 1D,G).

These NSC and NPC proteins were also evaluated in the PND5 piglet forebrain-SVZ. The forebrain-SVZ of newborn piglet was rich in nestin (Figure 3A) and musashi (Figure 3B). In contrast to the OB-SVZ, the forebrain-SVZ possessed darker immunostaining for nestin (Figs, 2E,3A), with the ependymal and subependymal layers differentially labeled (Figure 3A). The subependyma was more enriched in nestin immunoreactivity compared to the ependymal layer (Figure 3A, arrows). The forebrain-SVZ possessed more intense immunostaining for musashi compared to the OB-SVZ (Figures 2F, 3B). Moreover, numerous distinct musashi^+^ cells were present in the subependymal layer (Figure 3B). In subcortical white matter, putative migrating cells were nestin^+^ (Figure 3C) and Dlx2^+^ (Figure 3D). The latter marker suggests the presence of migrating newly born GABAergic interneurons [43]. Representative negative controls, when the primary antibodies were preadsorbed against recombinant nestin or Dlx2 protein prior to use, were blank (Figure 3E,F). Counts of these nestin^+^ and Dlx2^+^ cells in subcortical white matter revealed these immunopositive cells to be substantial populations of cells (Figure 3G). The numbers of nestin^+^ cells did not show maturation-related changes, while the numbers of Dlx2^+^ cells showed an age-related decline (Figure 3G). 

### The Piglet SVZ in Both Forebrain and OB Contain Numerous Newly Born Cells

**Figure 4:**
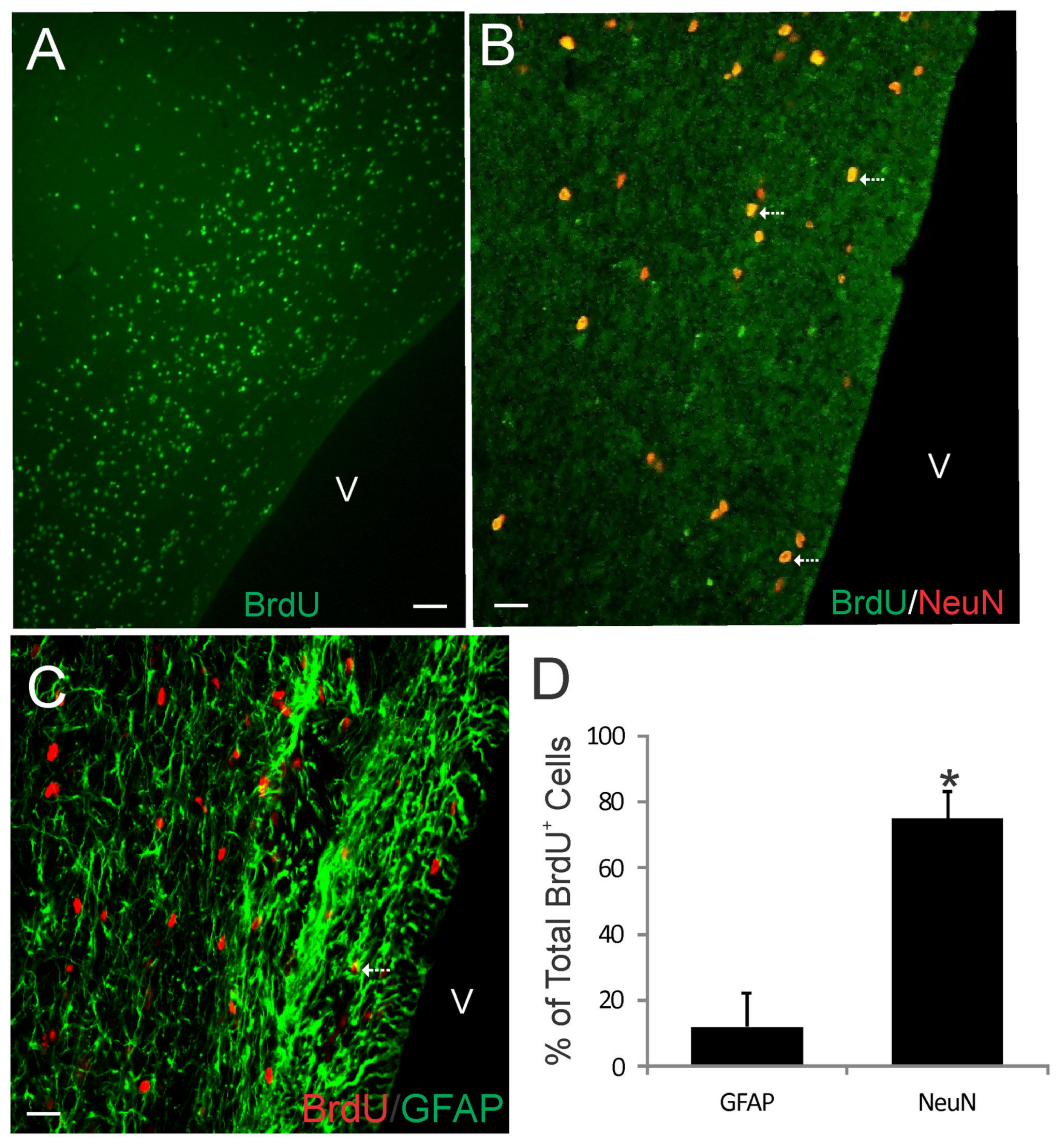
Bromodeoxyuridine (BrdU) cell proliferation tracking in piglet OB SVZ. **A** and **B**. Confocal microscope images showing that the OB contains numerous BrdU^+^ cells (A, green) and subsets of these cells are newly born neurons as shown by the colocalization (B, seen as yellow, arrows) with the neuron marker NeuN. **C**. Confocal microscope images showing the co-labeling (arrows) of the DNA synthesis marker BrdU (red) and glial fibrillary acidic protein (GFAP, green) in the OB SVZ. Some BrdU^+^ nuclei are associated with GFAP^+^ cytoplasm. Scale bars = 33 µm (A), 17 µm (B), 20 µm (C). **D**. Graph showing the proportions of the total BrdU^+^ cells that are either GFAP^+^ or NeuN^+^. Values are mean ± SD (n=4). Some BrdU^+^ cells were not positive for either marker. Asterisk denotes NeuN significant difference (p < 0.001) compared to GFAP.

**Figure 5:**
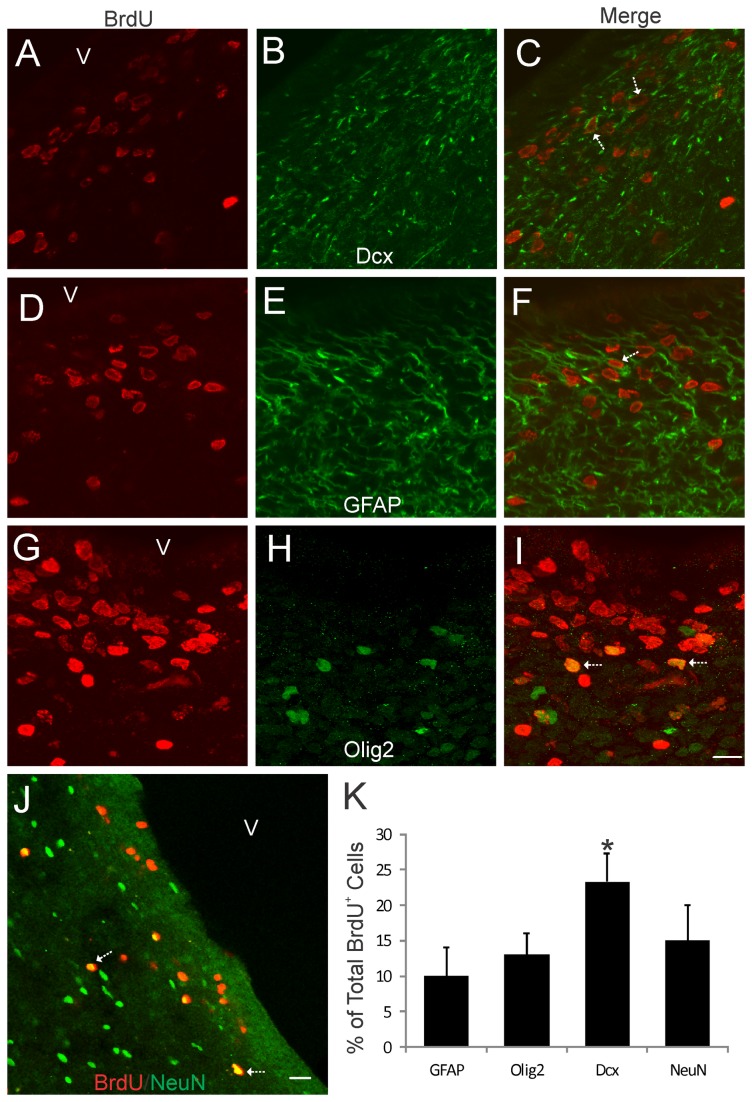
Bromodeoxyuridine (BrdU) cell proliferation tracking in piglet forebrain SVZ. Confocal microscope images showing that subsets of BrdU^+^ cells in the forebrain SVZ can be immunophenotyped (arrows) as cells positive for the neuroblast marker doublecortin (A-C, Dcx), the astrocyte marker GFAP (D-F), the oligodendrocyte marker Olig2 (G-I), and the neuron marker NeuN (J). The lateral ventricle is identified (v). Scale bars: (in I, applies to A-I) = 12 µm; (in J) = 23 µm. **K**. Graph showing the proportions of the total BrdU^+^ cells that are either GFAP^+^ Olig2^+^, Dcx^+^, or NeuN^+^. Values are mean ± SD (n=4). Asterisk denotes Dcx significant difference (p < 0.05) compared to GFAP, Olig2, and NeuN.

Proliferating cells in the piglet forebrain were studied using BrdU incorporation and confocal microscopy. BrdU labeling showed that the OB-SVZ (Figure 4A) and forebrain-SVZ (Figure 5A,D,G,J) in newborn piglets accumulate numerous newly replicated cells. This rapid accumulation of cells in OB after a single high BrdU pulse and short survival can be interpreted as resident proliferation and possible cells derived from the nearby RMS. No BrdU immunoreactivity was detected in control forebrain sections from piglets not injected with BrdU (data not shown). In OB, BrdU^+^ cells were immunoreactive for neuronal and astroglial markers (Figure 4B-D). The majority of newly born cells (~75% of total BrdU^+^ cells) found in the OB were neurons (Figure 4D). Relatively few BrdU^+^/GFAP^+^ cells (~10% of total BrdU^+^ cells) were observed in piglet OB (Figure 4D). Other studies have proffered astrocytes or another GFAP-expressing specialized cell type as the postnatal or adult NSC [44]. Our current observation in piglet OB is not inconsistent with the possibility GFAP^+^ cells act as NSCs in the mammalian OB. BrdU and GFAP co-labeling was also observed in ~10% of BrdU^+^ cells in the forebrain-SVZ (Figure 5D-F, K) as evidenced by the BrdU^+^ nuclei surrounded by a rim of GFAP^+^ cytoplasm. In some instances in the forebrain-SVZ, large GFAP^+^ processes radiated from cells with a BrdU^+^ nucleus (5F, arrow). Subsets of BrdU^+^ cells in the piglet forebrain-SVZ also co-localized with Olig2 (~12%), Dcx (~23%), and NeuN (~13%) (Figure 5A-C, G-K). Because our BrdU protocol had only a 24 h survival, it is likely that resident transit-amplifying cells [16,17] within the OB-SVZ and forebrain-SVZ and migratory routes are terminally dividing in situ and thus allowing cells time to express phenotypic markers. These data are consistent with results in the rodent OB [20,21].

### Identification of a lateral migratory stream of newborn neurons to piriform cortex

**Figure 6:**
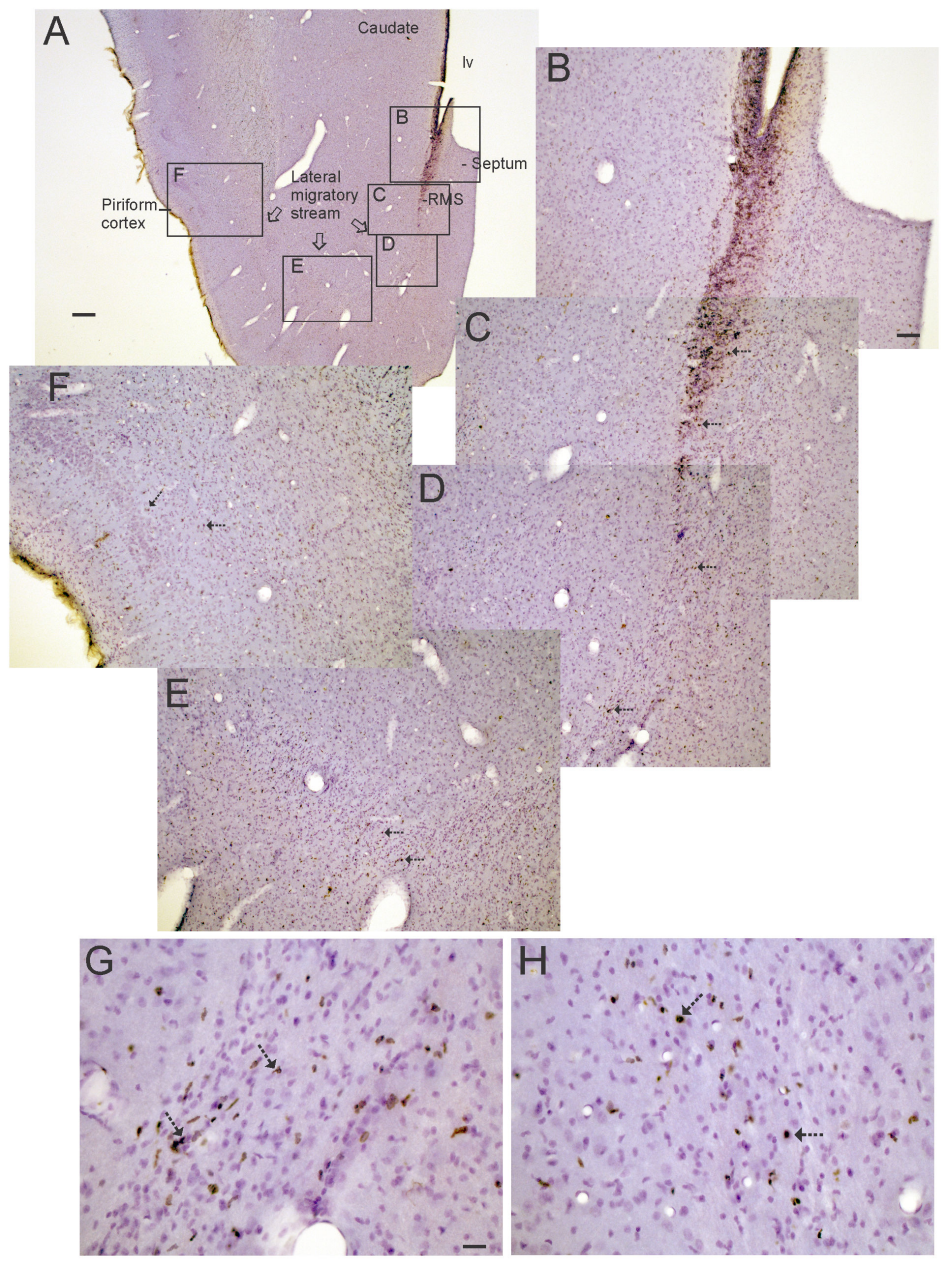
In vivo BrdU labeling in piglet forebrain reveals a new corridor of newly born cells named the lateral migratory stream. **A**-**H**. Images of a piglet forebrain section are shown with BrdU incorporation detected by the immunoperoxidase method (brown labeling) and counterstaining with cresyl violet. A low magnification image of piglet ventral forebrain is shown for perspective (A). Regions delineated by black rectangles in A are shown as higher magnification montage images in B-F that illustrate the distributions of BrdU labeled cells (hatched arrows, brown dots). Rostral migratory stream (RMS), and lateral ventricle (lv).**G and H**. Higher magnification images showing BrdU^+^ cell labeling (hatched arrows, brown nuclei) in the lateral migratory stream (G) beneath the striatum and in the piriform cortex (H). Unlabeled nuclei are pale violet. Scale bars: A = 300 µm, B (same for C-F) = 100 µm, G (same for H) = 25 µm.

**Figure 7:**
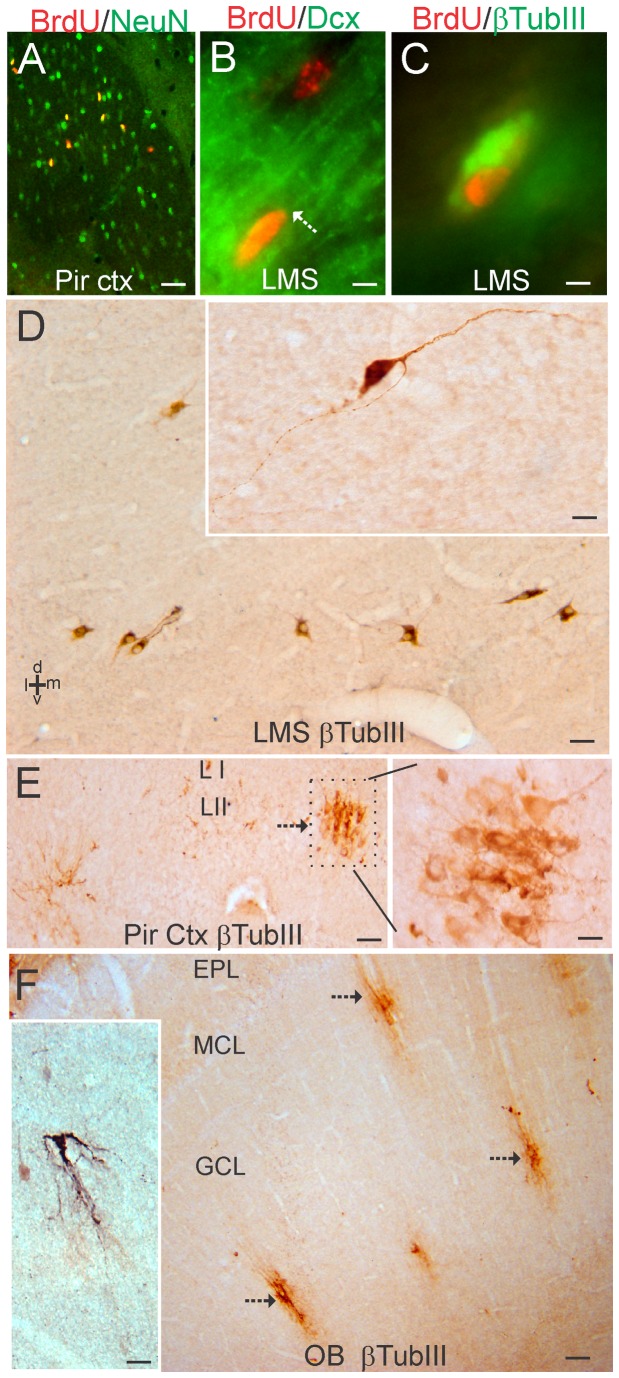
Characterization of the lateral migratory stream (LMS) of newly born neurons and identification of immature neurons in piglet forebrain. **A**. Confocal image showing the colocalization of BrdU (red) and NeuN (green) in subsets of neurons in the piriform cortex. Single-labeled neurons are green, single-labeled BrdU^+^ nuclei are red, and double-labeled neurons are yellow. **B**. Immunofluorescence showing the colocalization of BrdU (red) and doublecortin (Dcx, green) in the LMS. One Brdu^+^ cell (top cell) does not express Dcx, but another Brdu^+^ cell (bottom cell) is Dcx^+^ as seen by the yellow around the nucleus and the trailing Dcx-labeled process (arrow). **C**. Immunofluorescence showing the colocalization of BrdU (red) and β-tubulin III (βTubIII, green) in the LMS. **D**. Immmunoperoxidase staining for β-tubulin III (βTubIII) revealed a column of βTubIII^+^ immature neurons (brown cells) below the anterior striatum within the LMS. Inset shows a βTubIII^+^ LMS neuron with a fusiform morphology and leading and trailing processes. **E**. In piriform cortex, βTubIII^+^ immature neurons were found in cellular islands in layer II (arrow). Boxed area is shown at right at higher magnification and rotated 90°. **F**. In OB, βTubIII^+^ immature neurons (arrows) were found in the granule cell layer (GCL). These cells possessed extensive local vertical-oriented arborizations (inset) consistent with an interneuron morphology [46]. EPL, external plexiform layer; MCL, mitral cell layer. Scale bars = 25 µm (A), 5 µm (B), 4 µm (C), 25 µm (D), 8 µm (D inset), 32 µm (E), 12.5 µm (E right), 100 µm (F), 25 µm (F inset).

While analyzing the BrdU labeling in the piglet forebrain-SVZ, we found a group of BrdU^+^ cells (Figure 6) that resided ventrolaterally and appeared to split from the main SVZ-RMS transition that forms the column of cells migrating rostrally to the OB. These newly born cells (Figure 6G,H) tracked ventrolaterally beneath the striatum (Figure 6A-E) and were resident in the anterior lateral-ventral cerebral cortex, particularly the primary olfactory cortex (piriform cortex) (Figures 2A, 6A,F). We call this corridor of newly born cells that appear to provide neurons to the piriform cortex the “lateral migratory stream (LMS).” Many of the BrdU^+^ cells in piriform cortex were NeuN^+^ and, thus, were neurons (Figure 7A). NeuN is known to be expressed in mature neurons and newly postmitotic neurons [45]. In the ventrolateral corridor beneath the striatum BrdU^+^ cells were found to be positive for Dcx (Figure 7B) and β-tubulin III (Figure 7C), demonstrating that these newly born cells are immature neurons likely to be migrating in the LMS to the piriform cortex. 

### Localization of immature neurons in the piglet forebrain

A follow-up analysis of 5-day-old piglet forebrain sections stained for β-tubulin III revealed a highly selective distribution of immature neurons identified by this marker. Consistent with the BrdU/ β-tubulin III immunofluorescent co-labeling, β-tubulin III^+^ cells were found beneath the anterior striatum in the LMS (Figure 7D). In the piriform cortex, β-tubulin III^+^ neurons were found in distinct clusters in layer II (Figure 7E). In OB, subsets of β-tubulin III^+^ neurons with substantial local, vertically-oriented arborizations were found in the granule cell layer (Figure 7F). This morphology is consistent with OB granule cell layer interneurons [46].

### OB-NSCs/NPCs form neurospheres and are multipotent

**Figure 8:**
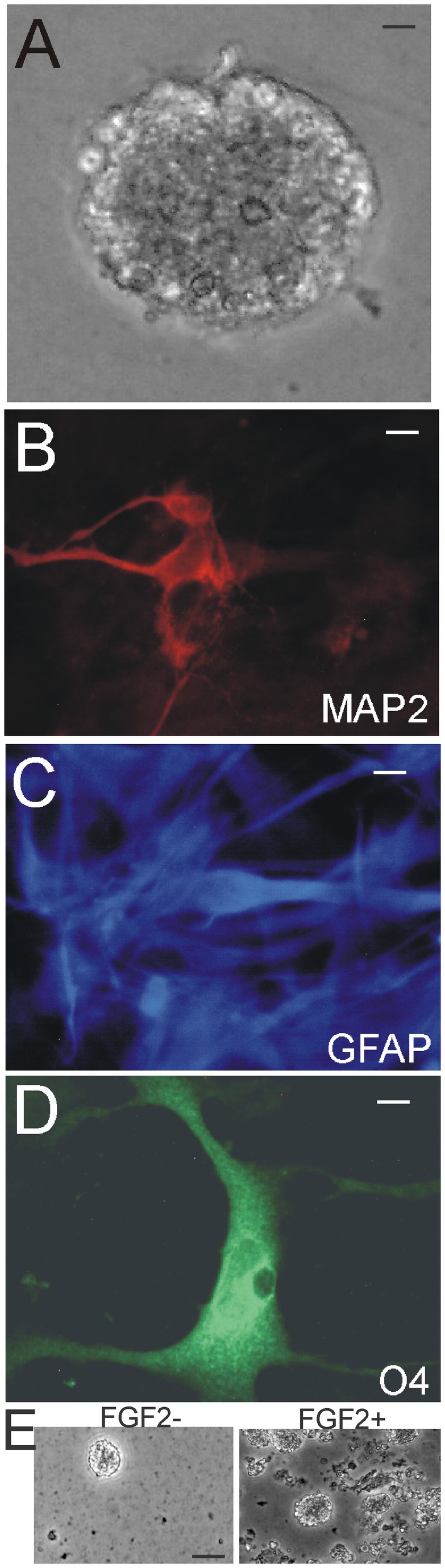
Newborn piglet OB-SVZ cells form multipotent neurospheres. **A**. Floating neurosphere formed by notch-1-immunopanned NSCs isolated from the OB. Scale = 12 µm. **B**-**D**. Attached neurospheres dispersed as monolayers are multipotent by differentiating into cells that are neurons identified by MAP2, astrocytes identified by GFAP, and oligodendrocytes identified by O4. **E**. Neurosphere density increased when medium was supplemented with FGF2. Scale bars = 12.5 (B), 20 (C), 20 (D), 50 (E) µm.

Periventricular tissues containing the OB-SVZ and the forebrain-SVZ regions from newborn piglet were precisely microdissected and used for cell culture experiments. SVZ- and OB-NSCs/NPCs were isolated by immunopanning for notch-1, cultured, and grown either as floating NSC neurospheres (Figure 8A) or as monolayers after the neurospheres were induced to attach (Figure 8B-D). OB-SVZ cells maintained a high capacity to self-renew in culture. The neurosphere-forming capacity of piglet OB-NSCs was determined in the presence of serum without mitogen supplements and in the presence of FGF2 without serum. OB-NSCs replicated in basic medium and the sphere densities increased rapidly in medium containing FGF2 or serum (Figure 8E). Attached OB-NSC spheres generated neurons (marked by MAP2), astrocytes (marked by GFAP), oligodendrocytes (marked by O4) (Figure 8B-D). Each antibody separately labeled cells with distinct morphologies. Piglet OB-NSCs could spontaneously generate cells of all 3 neural cell lineages when the same neurosphere cultures were maintained and passaged for over 90 days in culture. These data are consistent with results on the rodent OB [20,21,29]. 

**Figure 9:**
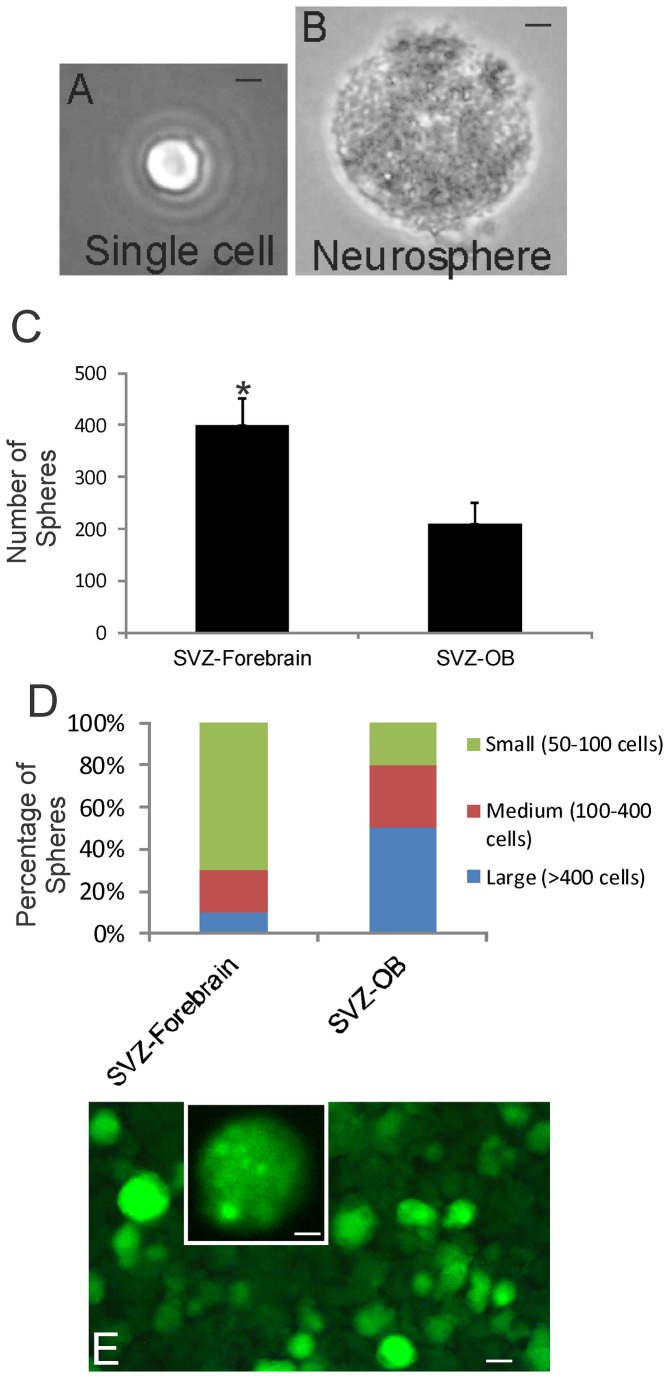
Single-cell clonal analysis of piglet OB- and forebrain-SVZ NSCs and genetic tagging with green fluorescent protein (GFP). **A**. A single cell isolated from a neurosphere derived from the OB SVZ. Scale bar = 12 µm. **B**. Secondary neurosphere formed from neurosphere-derived single cells. Scale bar = 12 µm. **C** and **D**. FGF2/EGF-stimulated growth assays on dissociated neurosphere cells isolated from the forebrain-SVZ and OB-SVZ. The number of secondary neurospheres formed (C, values are mean ± SD) and individual neurosphere cell number (D) were assessed. Asterisk denotes SVZ-forebrain significant difference (p < 0.01) compared to SVZ-OB. **E**. Numerous piglet OB-SVZ-derived neurospheres were robustly and stably transduced to express GFP by lentiviral gene transfer. Inset shows a single GFP-tagged neurosphere at higher magnification. Some individual cells can be seen in the sphere. Scale bars: 80 µm, 5 µm (inset).

We performed experiments (single-cell clonal analysis and chimeric analysis) to ensure the observations of clonogenicity and multipotency. For single-cell clonal analysis (Figure 9), OB-SVZ cells were isolated from newborn piglet and were dissociated completely and cultured on coated well plates. After 3 days in culture, only the floating spheres in the medium were collected, while the primary cells attached and were not collected. Individual spheres were dissociated mechanically or with trypsin into cell suspensions that were subjected to limited dilution. Neurosphere-derived cell suspensions were diluted in medium and aliquoted into 96 well plates. Each well was examined and the wells containing only one cell were identified (Figure 9A). Individual cells formed secondary neurospheres (Figure 9B). After 2-4 weeks, higher passaged spheres were formed and were transferred into coated wells for further expansion and for differentiation. The spheres were transferred to coated wells and were allowed to attach to the plate by reducing serum concentrations and omitting FGF2 in the medium. Spheres from some clones attached and differentiated as monolayers of cells. These attached spheres ranged in size from 21 to >400 cells. 

Forebrain-SVZ and OB-SVZ samples were trypsin digested and seeded on coated wells, and after 24 h, the medium containing floating cell spheres was transferred to non-coated wells for further expansion of highly proliferating sphere-forming cells, while attached cells, mostly primary cells, were not further evaluated. The rapidly dividing spheres were then further passaged. Neurospheres were trypsin digested and dissociated cells were immunopanned with antibody to notch-1 receptor. With immunopanning for notch-1^+^ cells, ~40% of forebrain-SVZ cells attached to the coated wells and ~ 15% of OB core cells attached to the coated wells. After washing wells to remove unbound cells, the attached cells were trypsinized (0.125% trypsin-EDTA for about 1 min) to detach them from the well. Cells were collected in growth medium again and transferred to new wells to expand for further analysis. The neurosphere-forming capacity of forebrain-SVZ NSCs and OB-SVZ NSCs (selected by notch-1 immunopanning) was different (Figure 9C,D). Forebrain-SVZ NSCs form smaller spheres and greater numbers of spheres than OB-SVZ NSCs (Figure 9C,D). These data demonstrate a difference between forebrain-SVZ and OB-SVZ-NSCs.

There is a possibility that primary dissociated piglet OB SVZ cells aggregate to form chimeric imposter-neurospheres during the first 3 days in culture. To avoid this possibility, neurospheres were grown and differentiated from isolated, individual cells immediately upon dissociation. In addition we mixed freshly dissociated piglet OB-SVZ cells with green fluorescent protein (GFP)-transfected OB-NSCs, and 3 days later looked for chimeric re-aggregations. None were found. 

### Lentiviral transfection of OB-SVZ NSCs

Neurosphere-forming OB-NSCs isolated from piglet were stably transfected with a GFP reporter gene using lentivirus (Figure 9E). Neurospheres were screened for GFP fluorescence after 48 h (~68% spheres were green at 48 h after exposure to lentivirus). Mock transfected neurospheres did not have green fluorescence. 

## Discussion

We conducted a histological and cell culture study of forebrain NSC/NPC niches in the newborn piglet brain. Our objective was to show the location, properties, and potential of NSCs/NPCs in this species. This interest in the piglet OB was inspired because this region in the postnatal brain could be a potential accessible source of NSCs/NPCs for autologous cell therapy in brain and spinal cord injury [20-22], and we use newborn piglets in a model of neonatal brain damage [23,25-27]. We demonstrate here that the newborn piglet forebrain-SVZ and OB-SVZ express markers for NSCs and NPCs, and these regions contain numerous replicating cells. The SVZ in the piglet OB covers an extensive circumferential territory. The cell culture characterization of the forebrain-SVZ and OB-SVZ showed that these regions contain resident cells that have self-renewal capacity and can generate clonal neurospheres that can be serially passaged and cryopreserved with maintained multipotentiality for generating neurons, astrocytes, and oligodendrocytes. Thus, cells in the newborn piglet OB-SVZ and forebrain-SVZ niches fulfill the criteria for multipotent NSCs and NPCs [47,48], and these cells can be frozen in a repository.

We isolated and cultured piglet forebrain-SVZ and OB-SVZ cells to study their NSC characteristics. We found that piglet OB-NSCs have a robust capacity to rapidly proliferate, form cell spheres, and differentiate into neurons, oligodendrocytes, and astrocytes. The self-renewal capacity and multipotency is maintained after 9 passages, cryopreservation and thawing, and after an additional 9 passages. Thus, piglet OB-SVZ contains multipotent NSCs [47,48]. To our knowledge, this in vitro characterization of forebrain and OB NSC niches in the newborn piglet brain is novel. The postnatal rodent OB has been used to isolate and characterize in vitro NSCs [19-22]. These earlier studies showed that adult rat and mouse OB core cells have the cardinal features of NSCs by population and formal clonal analyses. The human OB also contains resident NSC/NPCs [20,49,50]. We now show that piglet OB-SVZ cells display long-term self-renewal capacity and the ability to generate clones, express nestin, and differentiate in vitro into neurons, astrocytes, and oligodendrocytes. Isolated OB-SVZ cells replicated vigorously to generate primary neurospheres that were capable of generating secondary- and higher-passaged spheres. The proliferation capacity of OB-SVZ cells in vitro is consistent with our in vivo BrdU results showing robust BrdU incorporation. OB-SVZ neurospheres could be genetically tagged permanently with GFP, and GFP-tagged neurospheres could be cryopreserved, thawed, and subsequently passaged. GFP-tagged neurosphere-forming cells will be a valuable tool for cell tracking in future transplantation experiments in models of neonatal/pediatric CNS injury. 

Three previous studies have documented the isolation, culture, and characterization of neural precursor cells from porcine fetal brain [51-53]. One study isolated, from cerebrum of 60-day pig fetuses, and grew neurosphere-forming cells in the presence of FGF2 and EGF and demonstrated, by RT-PCR, expression of NPC and glial markers as well as Sox2 [51]. The porcine cells were notable for having many characteristics of human NPCs [51]. Another study isolated, from forebrain SVZ of 65-80 day pig fetuses, and grew neurosphere-forming cells in the presence of FGF2 and EGF over long-term during which neurospheres spontaneously differentiated into the 3 primary neural cell lineages [52]. A more recent study used fetal day 28-30 pigs to isolate and propagate in culture ventral mesencephalic NPCs [53]. Thus, our study builds on the value of the swine brain in NSC/NPC research and is the first to demonstrate the isolation, propagation, and differentiation of large numbers of multipotent NSCs from the postnatal piglet forebrain-SVZ and OB-SVZ.

The cellular composition of the forebrain neurogenic niche has been studied extensively in adult rat and mouse, but scant information has been available on the forebrain neurogenic niche in large quadrupeds. The ependymal-subependymal region of the rodent forebrain-SVZ contains at least 4 different cell types (types A, B, C and E) divisible by morphology and molecular markers [16]. Historically, the primacy of the NSC in rodents has been bestowed upon two different cell types (the astrocyte and the ependymal cell) [30,54,55]. A layer of ependymal cells (type E cells) separates the SVZ from the ventricular cavity. Formation of multipotent neurospheres from ependymal (type E) cells was reported [30]. However, using a microdissection method, van der Kooy’s group showed that ependymal and subependymal cells in the forebrain SVZ of adult male mice have different characteristics, with only subependymal cells forming multipotent neurospheres [32]. Multipotent progenitor cells can also be astrocytes [54] that can grow as NSCs in culture [55] and can generate EGF- and FGF-responsive neurospheres [44]. We have found EGF- and FGF-responsive cells isolated from the piglet forebrain-SVZ and OB-SVZ that can generate neurospheres. The Steindler group found that ciliated ependymal cells are unipotent, giving rise only to glia, while forebrain-SVZ astrocytes (type B cells) form multipotent neurospheres that produce both neurons and glia in immature and adult mice [44]. This evidence identified in mouse brain the astrocyte or a specialized GFAP-expressing type B cell that divides slowly as the forebrain-SVZ NSC and the primary precursor of new neurons in the forebrain-SVZ. Type A cells are migrating, Dcx^+^ young neurons (neuroblasts) that form columns of cells that are ensheathed by astrocytes (type B cells) [16]. Rapidly dividing, cytosine arabinoside-sensitive neural precursor cells (type C cells, also called transit-amplifying cells) are generated from type B cells and form clusters next to the columns of chain-migrating neurons [16]. Type C cells generate neuroblasts (type A cells) and are thought to be the cells robustly detected by ^3^H-thymidine or BrdU, but type A and B cells replicate as well in rodents [16,44]. Cell types A, B, and C could all be involved in the generation of new neurons; however, isolated type A cells do not appear to self-renew in vitro, while rodent type B and C cells appear to generate large colonies of young neurons [55]. We observed numerous BrdU^+^ cells in the piglet forebrain-SVZ and OB-SVZ after a single BrdU pulse and short survival. In OB, most BrdU^+^ cells were NeuN^+^, demonstrating that they are newly born neurons most likely to be OB- SVZ-generated, rather than being derived from the RMS. NeuN expression in neurons coincides with cell cycle exiting and initiation of terminal differentiation [45]. A small proportion (~10%) of the BrdU^+^ cells in the OB-SVZ was GFAP^+^ and may represent resident NSCs. A similar minor presence of subsets of BrdU^+^/GFAP^+^ cells in piglet forebrain subependymal layer supports this idea. However, the identity of many (~40%) BrdU^+^ cells in the newborn piglet forebrain SVZ remains uncertain. 

The forebrain-SVZ and OB-SVZ NSC niches in newborn piglet brain are different in composition. Notch-1^+^ cells from the piglet forebrain-SVZ generated greater numbers of smaller (fewer cells) neurospheres than OB-SVZ counterparts which were generally larger (more cells). Differences in neurosphere-forming and differentiation capacities have been shown for multipotent NSCs residing in the SVZ, RMS, and OB in adult mice [19]. Histology revealed that the forebrain-SVZ and OB-SVZ in piglet show prominent differences in the numbers of BrdU^+^ cells that were NeuN^+^. One other study has reported on the abundant BrdU labeling in the 1-week-old piglet forebrain-SVZ, but the OB was not studied [56]. We also show that the localizations of nestin and musashi in the ependymal and subependymal layers of these two SVZ niches were different. Both nestin and musashi appeared more enriched in forebrain-SVZ compared to the OB-SVZ. Thus, although multipotent NSCs exist throughout the mammalian SVZ-OB neurogenic system, our findings in piglet demonstrate that the anatomical components of this system are not homogeneous and display inherently different histological and developmental properties. This information is relevant to the reasoning behind choosing the forebrain SVZ or the OB SVZ for sources of cells for transplantation therapies. 

Our in vivo BrdU labeling experiments in piglet identified a unique group of newly born neurons in the newborn brain that to our knowledge has not been observed before in any species. While examining the anterior forebrain-SVZ and the emergence of the RMS, some of the newly born cells were found to branch from the main thoroughfare to the OB and form a separate corridor of cells apparently migrating laterally beneath the ventral striatum. We designate this group of cells the “lateral migratory stream (LMS).” Double-labeling demonstrated that BrdU^+^ cells within the LMS were positive for Dcx and β-tubulin III, thus identifying these cells as migrating immature neurons [57,58]. The BrdU^+^ cells congregated in the vicinity of the primary olfactory cortex, and double-labeling revealed that the newly born cells were neurons. 

The current conceptual dogma generally maintains that cells generated in the SVZ migrate rostrally in the RMS to the OB to continually populate this region with interneurons [16]. This conceptual framework is consistent with our finding that the piglet OB granule cell layer contains subsets of β-tubulin III^+^ immature neurons with robust local, vertical-oriented arborizations consistent with newly born interneurons [46]. However, we also find in piglet that newly generated cells can depart from the anterior part of the RMS and travel laterally to the piriform cortex. Other non-RMS routes for the migration of SVZ-generated newborn neurons have been found in other species. One migratory pathway from the SVZ found in adult monkeys supplies neurons to the amygdala and nearby cortical regions [59]. Another study discovered a ventrocaudal migratory stream of newly born cells to the piriform cortex in early postnatal mice [60]. In a study of the human brain, a “lateral migratory stream” was not reported, but a medial migratory stream that diverged from the RMS and traveled to the ventral medial prefrontal cortex was observed [61]. Considering the roles of the piriform cortex and OB in the sensory synaptic plasticity olfaction [46,62], a continual supply of newly born neurons to these regions may be related to dynamic isochronic networking of neurons and functional plasticity within the olfactory system. The distribution of β-tubulin III^+^ immature neurons seen as islands in layer II of piriform cortex (Figure 7E) may be related to functional linkage of piriform cortical neurons with OB microdomains modulated by similar birth-date interneurons. Our observations on the postnatal plasticity of the OB and piriform cortex could also be relevant to the well documented finding that loss of olfaction is an early prominent feature of prodromal Parkinson’s disease occurring before motor symptoms emerge [62].

Our study of NSCs and NPCs in the piglet OB reveals developmental changes in the composition of this neurogenic niche. The levels of Dcx and PSA-NCAM proteins were much lower in OB-SVZ extracts from older piglets compared to newborn piglets. The developmental change in Dcx detected by western blotting was mirrored by the immunohistochemical observation showing an attenuation of immunoreactivity at PND30 compared to PND5 (Figure 2G). A maturation-related reduction of migrating Dcx^+^ and PSA-NCAM^+^ newborn neurons appears to occur in the brain of human children [61]. However, an interesting finding in piglet OB-SVZ is the maintained expression of Dlx2 and the up-regulated expression of musashi with maturation (Figure 1). Given that Dlx2 is a transcription factor that functions in cell fate determination [35] and musashi is a NSC marker that functions in asymmetrical cell division and NSC renewal [38], we conclude that their presence in the OB-SVZ signifies the sustained presence of resident NSCs in the piglet OB. Our cell culture results, including the notch-1 immunopanning, single-cell clonal analysis and the neurosphere formation assay, are consistent with a resident population of NSCs within the OB. We found previously the presence of NSC markers in the human OB [20]. Thus, the OB is a reservoir of NSCs accessible for cell transplantation therapy for brain injury. 

An emerging hope exists for regenerative medicine in the treatment of perinatal brain injury [1,2]. A variety of studies, all performed in rodents, have documented the therapeutic benefits of different rodent- and human-derived cells in models of HI or excitotoxic brain injury [4-9]. However, if allogenic human cell lines are ultimately implemented clinically, long-term immunosuppression will have to be done. Immunosuppression is achieved clinically after transplantation by treatment with cyclosporine A or tacrolimus, but these drugs can induce numerous side effects, including hypertension, diabetes, nephrotoxicity and neurotoxicity, in as many as 40% of patients [63]. Autologous NSCs/NPCs and induced pluripotent stem cells would have a tremendous advantage in this regard for the avoidance of the ramifications of chronic immunosuppression and to personalize treatment for infants and children. Our results provide proof that the piglet OB-SVZ is a reservoir of NSCs and NPCs. The OB is significant because this region is more accessible than other intracranial sources of NSCs/NPCs. Neurosurgical approaches are established for exposing the human OB [64,65]. It is noteworthy that the piglet OB has an open ventricular cavity similar to that reported for the OB in human [66]. Trans-plantation of rodent OB NSCs/NPCs have shown efficacy in adult rodent models of spinal cord injury [21,22]. Our new information has translational significance in that it provides a rationale to access the OB as a source of autologous cells for transplantation therapy in preclinical large animal models of neonatal brain injury [23-27].
